# Adipose Tissue Wasting as a Determinant of Pancreatic Cancer-Related Cachexia

**DOI:** 10.3390/cancers14194754

**Published:** 2022-09-29

**Authors:** Seok-Yeong Yu, Yi Luan, Rosemary Dong, Amirhossein Abazarikia, So-Youn Kim

**Affiliations:** 1 Olson Center for Women’s Health, Department of Obstetrics and Gynecology, College of Medicine, University of Nebraska Medical Center, Omaha, NE 68198, USA; 2 Fred and Pamela Buffett Cancer Center, University of Nebraska Medical Center, Omaha, NE 68198, USA

**Keywords:** pancreatic cancer, cachexia, adipose tissue wasting, adipocyte

## Abstract

**Simple Summary:**

Pancreatic cancer (PC) is one of the deadliest cancers in the US. The poor prognosis of PC is related to diagnostic delay and the presence of unintended weight loss (cachexia) that commonly presents in PC patients even before diagnosis. However, the current understanding of how PC mediates cachexia is limited, and there are few treatments clinically available for cachexia. Based on the current literature, we demonstrate that PC-related cachexia primarily results from the wasting of adipose tissue, once thought to be merely a storage depot but now appreciated as an instrumental metabolic organ in the body. In addition, poor survival in PC patients was found to be associated with adipose tissue loss at diagnosis and during treatment. Therefore, identifying potential mediators and molecular mechanisms underlying adipose tissue loss would promise to pave the way for the development of effective interventions for PC-related cachexia

**Abstract:**

Pancreatic cancer (PC) is the third leading cause of cancer-related death in the US, and its 5-year survival rate is approximately 10%. The low survival rates largely stem from diagnostic delay and the presence of significant adipose tissue and muscle wasting, commonly referred to as cachexia. Cachexia is present in nearly 80% of PC patients and is a key cause of poor response to treatment and about 20% of death in PC patients. However, there are few clinical interventions proven to be effective against PC-related cachexia. Different cancer types feature distinct secretome profiles and functional characteristics which would lead to cachexia development differently. Therefore, here we discuss affected tissues and potential mechanisms leading to cachexia in PC. We postulate that the most affected tissue during the development of PC-related cachexia is adipose tissue, historically and still thought to be just an inert repository for excess energy in relation to cancer-related cachexia. Adipose tissue loss is considerably greater than muscle loss in quantity and shows a correlation with poor survival in PC patients. Moreover, we suggest that PC mediates adipose atrophy by accelerating adipocyte lipid turnover and fibroblast infiltration.

## 1. Introduction

Pancreatic cancer (PC) is the third leading cause of cancer-related death in the US [1]. Pancreatic ductal adenocarcinoma (PDAC) is the most prevalent and aggressive type of PC, with a 5-year survival rate approaching approximately 10% [2]. Surgical resection when diagnosed as being respectable offers the best prognosis [3]. However, most patients are diagnosed at locally advanced or metastatic stages which are considered unresectable [4]. More than 80% of PC patients also present an unintended, significant weight loss, commonly referred to as cachexia, which worsens prognosis and accounts for 20% of PDAC-related deaths [5,6], although obesity appears unrelated to survival [7,8]. According to international consensus, the onset of cachexia is described as more than 5% weight loss for patients with BMI ≥ 20 kg/m^2^, more than 2% weight loss in underweight patients (BMI < 20 kg/m^2^), or more than 2% weight loss and sarcopenia [9,10]. PC-related cachexia often presents before PC diagnosis and exhibits adipose tissue and skeletal muscle wasting, with a concomitant presence of systemic inflammation [11]. With progressive cachexia, PC patients experience poorer quality of life, reduced physical performance, and lower response to and tolerance of chemotherapy [12]. Therefore, managing cachexia would benefit patients by improving their prognosis and response to chemotherapy. However, there are currently few treatments available against cachexia for PC patients [13]. Different cancer types exhibit distinct transcriptome and secretome characteristics [14], and, thus, the mechanisms for developing cachexia are expected to be different by cancer type. Hence, understanding the affected tissues and mechanisms for PC-related cachexia is imperative for developing relevant interventions for cachexia in PC patients. Notably, existing studies that characterized weight loss in PC patients have demonstrated that adipose tissue loss is considerably greater than skeletal muscle loss in quantity, suggesting adipose tissue as the most affected tissue that primarily mediates PC-related cachexia. Therefore, this review focuses on our current understanding of PC-related cachexia, with an emphasis on adipose tissue loss and cellular mechanisms underlying adipose tissue loss.

## 2. Adipose Tissue Wasting as a Critical Determinant for Cachexia and Poor Prognosis in Pancreatic Cancer Patients

Cachexia at diagnosis and during chemotherapy is a predictor of poor survival in PC patients [15]. Nemer et al. [16] reported that approximately 90% of PDAC patients presented weight loss at diagnosis, which was measured by BMI, with 75% of them having more than 5% weight loss. Additionally, greater than 10% of weight loss at diagnosis, which was present in about 40% of newly diagnosed PDAC cases showed a 1.7 times higher risk of death. When body composition was measured by computed tomography (CT) during chemotherapy, Kays et al. [17] demonstrated that fat-only loss increased the risk of death by 5 times, and fat and muscle loss had 2 times higher risk of death in the patients with advanced PDAC when compared to patients who had no wasting.

Adipose tissue loss primarily contributes to weight loss in PC patients and leads to poor prognosis in PDAC patients [15,18]. Wigmore et al. [19] reported that patients with unresectable PDAC and no history of treatment lost 23% of total fat mass and 7.5% of lean mass on average from the time of diagnosis until death. Tan et al. [20] also observed about 45% total adipose tissue loss and 5.5% skeletal muscle loss in PC patients in advanced stages, primarily stages III and IV over 135 days since diagnosis. In the studied population, subcutaneous and visceral adipose tissue (SAT and VAT) loss similarly contributed to the total fat loss [20]. Consistently, CT scans from more than 1-year follow-ups by Di Sebastiano et al. showed 13.7% VAT and 2.24% skeletal muscle loss per 100 days given the weights at the first scan in PC patients [21]. Interestingly, when survival was stratified by specific tissue wasting, VAT loss predicted poorer survival in the PC patients, whereas changes in skeletal muscle did not influence survival rates [21]. Supporting this observation, Nakano et al. [22] demonstrated that more than 19% VAT loss increased the risk of death up to 2.4 times when compared to less than 19% VAT loss in the patients with unresectable PC, although no difference in overall survival rates was observed by skeletal muscle loss stratified by 2%. However, sarcopenia in obese PC patients is shown to worsen prognosis [20,23], suggesting that adiposity is a key modifiable risk factor for prognosis in PC patients.

Accumulated evidence indicates that malassimilation and tumor size are risk factors for skeletal muscle loss in PC patients as demonstrated by improved maintenance of skeletal muscle mass through enteral tube feeding and tumor resection [24,25,26]. However, the mechanisms underlying adipose tissue loss have remained unclear. Enteral tube placement or nutritional supplement was not effective in attenuating adipose tissue loss rates in PC patients [21,26]. Moreover, Sandini et al. [25] demonstrated that substantial VAT loss still occurred in patients with locally advanced pancreatic cancer during neoadjuvant treatment and was not mitigated by tumor resection in the patients. The presence of diabetes appears to be related to VAT loss in PC patients, showing that VAT loss rates were 2 times higher in PC patients with diabetes than those without diabetes [21]. As it is evidently observed that diabetes is related to elevated levels of blood fatty acids because of tissue insulin resistance [27], it is expected that adipose tissue loss is more likely severe in PDAC patients with diabetes versus those without diabetes. Histologically, we have demonstrated that high infiltration of fibrosis was detected in the area enriched with small adipocytes [28]. Therefore, observational evidence indicates that mechanisms leading to adipose tissue loss in PC patients would be chronically affected and/or related to glucose intolerance, while further studies are required to address if surgical removal and nutritional support would render benefits in mitigating adipose tissue loss.

## 3. Decreased Intake and Promoted Adipose Lipid Turnover to Exacerbate Adipose Tissue Loss in Pancreatic Cancer Patients

**Figure 1:**
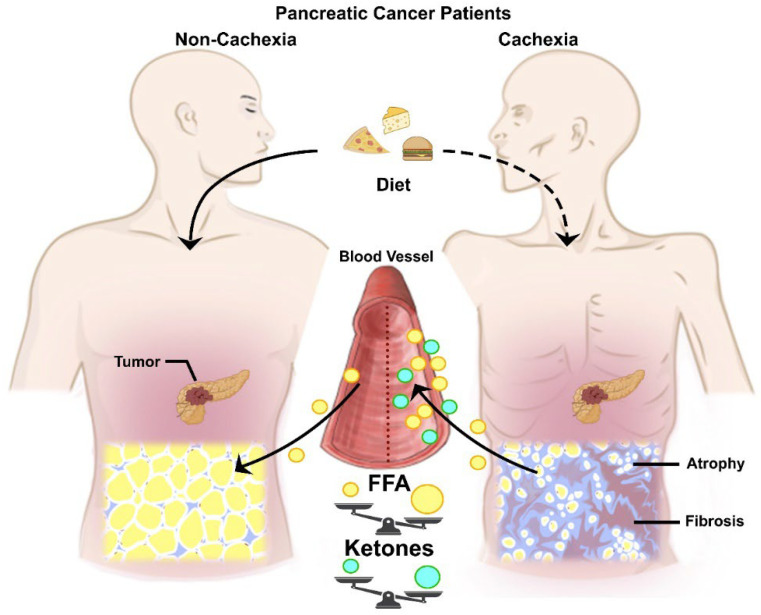
Schematic summary of altered fatty acid mobilization in pancreatic cancer patients with cachexia. Pancreatic cancer-related cachexia is primarily contributed by adipose tissue loss. Pancreatic cancer patients with cachexia appear to have lower dietary intake but have higher free fatty acid and ketone levels in circulation when compared to pancreatic cancer patients without adipose tissue wasting. Adipose atrophy is characterized by high infiltration of fibrosis and small size of adipocytes.

As PC stage advances, adipose tissue loss becomes increasingly prominent and mainly mediates cachexia in PC patients [29,30]. Evidence from studies that investigated blood lipid profiles in PC patients provides a potential physiological insight into adipose tissue loss. The lipoproteins serve as transport vehicles for lipids such as phospholipids, cholesterols, and fatty acids through circulation [31]. The lipoproteins are classified by density and size into four major classes and include chylomicron, very-low-density lipoprotein (VLDL), low-density lipoprotein (LDL), and high-density lipoprotein (HDL) [32]. Macasek et al. [33] demonstrated that when compared to healthy individuals, PC patients exhibited no changes in total cholesterol and LDL levels but had a significant elevation of free fatty acid levels and reduction in HDL levels in circulation. As not all PC patients present cachexia, when PC patients are grouped by the presence of cachexia, Fujiwara et al. [34] found that total blood cholesterol and LDL levels are notably lower in stage IV PC patients with cachexia than weight-stable PC patients. HDL levels were not statistically different between the groups of stage IV PC patients with or without cachexia [34]. Profiles of fatty acid composition in plasma lipids from PC patients further reveal that newly diagnosed PC patients with no history of treatment had elevations in several fatty acids such as palmitic acid (16:0), stearic acid (18:0), and oleic acid (18:1n-9) and had lower levels of linoleic acid (18:2n-6) and α-linolenic acid (18:3n-3) compared to healthy individuals [35]. Consistent observations have been made by Macasek et al. [33] that PC patients had higher levels of palmitic acid, stearic acid, and oleic acids and lower levels of linoleic acids in the plasma lipids than healthy subjects. Palmitic acid and oleic acid are the primary saturated and monounsaturated fatty acids in diets and are abundantly found from adipose tissue [36]. Unlike palmitic and oleic acids, linoleic acid and α-linolenic acid are essential fatty acids that should be provided from diets as the body cannot synthesize them [37]. Therefore, reduced levels of linoleic acid and α-linolenic acid but elevated levels of palmitic acid and oleic acid in PC patients’ plasma lipids indicate reduced dietary intake of lipids and increased fatty acid release from adipose tissue (Figure 1).

**Figure 2:**
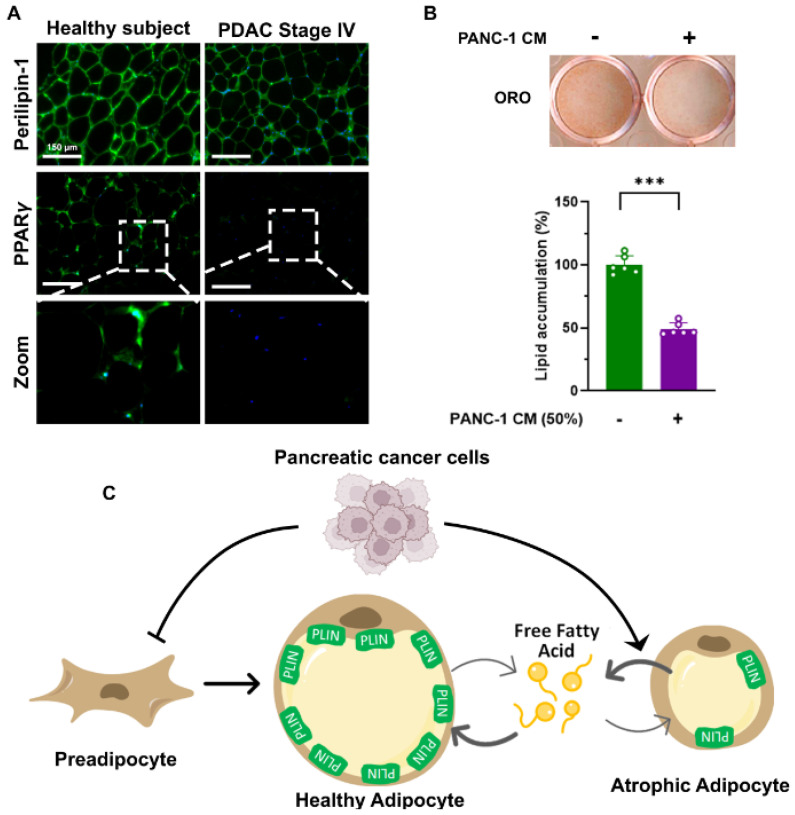
Proposed mechanism for adipose tissue atrophy related to pancreatic cancer. (**A**) Immunofluorescence assay of visceral adipose tissue with Perilipin-1 and PPARγ from healthy donors and PDAC stage IV patients (*n* = 5 per group). Blue, DAPI; Green, Perilipin-1 and PPARγ. (**B**) 30 min pre-exposure of PANC-1 conditioned media (PANC-1 CM) suppresses 3T3-L1 adipocyte differentiation evaluated by Oil Red O (ORO) staining. ***, *p* < 0.001. (**C**) Pancreatic cancer suppresses adipocyte differentiation and promotes lipid hydrolysis in existing adipocytes by reducing adipogenic genes for lipid storage. Such alterations in adipose tissue constitute the development of adipocyte atrophy and fibrosis. PLIN, perilipin-1.

Moreover, omega-3 fatty acid levels in plasma phospholipids are negatively correlated with the stage of PC, whereas palmitic acid and oleic acid levels were similar across the different stages of PC [33]. These observations additionally suggest that PC would accompany fatty acid release from adipose tissue at the early stage while gradually suppressing dietary fat intake with advancing stages in the PC patients. Indeed, a retrospective cohort study that characterized temporal changes in fat and lean mass for 60 months before PDAC diagnosis showed that weight loss precedes PDAC diagnosis [29]. Subcutaneous adipose tissue (SAT) loss appeared to occur in the early stage of weight loss, followed by VAT and lean mass loss in the PDAC patients until diagnosis [29]. However, the reason why SAT loss precedes VAT loss in PDAC patients is unclear. It has been shown that visceral adipocytes are more sensitive to catecholamines but less responsive to insulin, making them more lipolytic than subcutaneous adipocytes [38]. It is possible that fat loss in PDAC patients may be largely mediated by basal lipolysis, not hormone-mediated lipolysis. Perilipin-1 (PLIN1) is preferentially expressed in adipocytes, and its unphosphorylated form coats lipid droplets to prevent triglyceride hydrolysis [39]. In support of this, we demonstrate that when compared to VAT from healthy individuals, the fluorescent intensity of perilipin-1 is significantly reduced in VAT from PDAC stage IV patients (Figure 2A).

Concomitantly, the fluorescent signal of peroxisome proliferator-activated receptor gamma (PPARγ), which is the well-known transcription factor for lipogenic genes including PLIN1 in adipocytes, darkens in cytoplasmic and nuclear parts of adipocytes in VAT from PDAC stage IV patients (Figure 2A). Thus, suppressed expression of perilipin-1 in PDAC patients would be expected to accelerate triglyceride turnover favoring toward liberating free fatty acids and promoting adipose tissue loss as phenotypically observed in *Plin* knockout mice and in individuals with polymorphism at the *Plin* locus who exhibited lower body weight [40,41].

In PDAC patients, high blood fatty acid levels largely mediated from adipose tissue would be the consequence of the whole-body metabolic shift to adapt to the low-carbohydrate environment in which dietary intake and glucose availability to normal tissues decrease as cancer cells grow. Correspondingly, blood ketone bodies are shown to be significantly elevated in PDAC patients than in normal and even diabetic subjects [42]. Increased fatty acid oxidation by tissues yields acetyl-CoA as the final product which will be synthesized into ketone bodies such as acetoacetate and 3-OH butyrate through ketogenesis primarily in the liver [43]. The by-products are then metabolized back to acetyl-CoA through ketosis to run the tricarboxylic acid cycle and electron transport system in extrahepatic tissues. Given the high free fatty acid and ketone body levels in PC patients, it is expected that as PDAC tumor increases glucose utilization, normal tissues increase the demand for the use of fatty acids as an energy source. Interestingly, ketone bodies have been shown to decrease glucose metabolism and reduce cell viability in multiple in vitro human PDAC cell lines [44]. In mice, ketogenic diets diminished PDAC tumor growth with concomitant mitigations of weight and muscle loss and sensitized tumor to cytotoxic chemotherapy [44,45]. However, it is important to note that the tumor-suppressing effects of ketogenic diets are inconsistent across literature [45,46]. Therefore, a potential anti-tumorigenic mechanism which ketogenic diets act through remains to be addressed.

## 4. Pancreatic Cancer-Related Adipose Tissue Remodeling to Promote Adipocyte Lipid Turnover and Fibrosis

The glucose analog [18F]-Fluoro-2-deoxy-2-d-glucose (18F-FDG) is a widely used positron emission tomography (PET)/CT radiotracer, and the tissue distribution pattern of FDG allows the tumor detection and measurement of tissue glucose metabolic activity [47]. FDG-PET-CT imaging in PDAC patients has revealed that FDG accumulation in primary tumors increased with the stage of PDAC and the presence of distant metastasis. FDG uptake by SAT was decreased in stage III and IV patients than in stage I and II patients [48]. VAT showed increased FDG uptake as VAT thickness decreased in the PDAC patients [48]. These findings indicate that, as PDAC tumor grows and spreads, glucose availability to SAT would decrease, whereas glucose deposition increases in VAT area with VAT mass decreased in PDAC patients. However, it is important to note that tumor infiltration into adipose tissue in the pancreatic tumor section is frequently observed, and adipocytes in front of invasive cancer cells are characterized by smaller cell sizes than those in distal parts [49,50]. Therefore, it should be taken into consideration that the cancer cells spreading to the omentum and infiltrating into VAT might increase FDG uptake in VAT, while the adipocyte size of SAT and VAT would decrease in PDAC.

PC cells have been shown to decrease adipocyte size by increasing the hydrolysis of triglycerides. Co-culture with the human PC cell line, PANC-1 or MIA PaCa2, significantly reduced the size of lipid droplets and increased glycerol release from differentiated mature 3T3-L1 adipocytes which are a well-characterized in vitro white adipocyte model [50,51,52]. Consistently, glycerol release from 3T3-L1 adipocytes or free fatty acid release from differentiated adipose-derived mesenchymal stem cells was augmented by co-culture with or conditioned media from PC cells isolated from genetically engineered PDAC mouse model, KrasG12D; Trp53R172H;Pdx1-Cre (KPC) [49,53]. Gene expression analysis showed that co-culture with PANC-1 and MIA PaCa2 cells significantly inhibited the mRNA expression of lipases (*Hsl* and *Atgl*) with a concomitant suppression of HSL at the protein level in 3T3-L1 adipocytes [50,54]. In addition, transcriptome analysis demonstrated that the genes for de novo fatty acid synthesis, triglyceride synthesis, and related upstream nuclear receptor *Pparg* were downregulated in adipocytes co-cultured with PANC-1 [50]. Insulin sensitivity was suppressed by the co-culture with PANC-1 as demonstrated by decreased Akt phosphorylation by insulin stimulation and reduced *Glut4* mRNA expression in 3T3-L1 adipocytes [50]. PPARγ has been recognized as a key nuclear receptor for various lipogenic genes and adipocyte metabolism [55]. The failure of PPARγ transcriptional activation in differentiated 3T3-L1 adipocytes characterizes reductions in glucose and fatty acid uptakes but promoted basal lipolysis [56]. Moreover, PPARγ inactivity downregulates the mRNA expression of its target genes for triglyceride synthesis and lipolysis including *Plin* and *Hsl* in the cells, and such disturbed transcriptional regulations are consequently reflected into reducing energy intake but promoting basal lipolysis [56,57]. Suppressed PPARγ protein and *Pparg* mRNA expression in differentiated 3T3-L1 adipocytes by co-cultured with human PC cell lines has been demonstrated by multiple studies [50,51,54]. We reported suppressed mRNA expression levels of *Pparg* and its downstream genes (*Adipoq* and *Fasn*) in epididymal white adipose tissues (eWAT) from S2–013 orthotopic PC mice when compared to those from the sham group [28]. Therefore, decreased adipocyte size in PC would be mediated by decreased *de novo* lipid synthesis coupled with promoted basal lipolysis (Figure 2A) and be attributed to the suppression of PPARγ expression in adipocytes as phenotypically observed in *Pparg*-mutant adipocytes [57]. However, it is unknown how PC suppresses adipocyte *Pparg* expression and whether exogenous PPARγ ligands would restore such metabolic dysregulations in adipocytes.

Adipocyte browning is suggested to contribute to adipose tissue wasting in different cancer cachexia models such as lung cancer, liver cancer, and PC, all of which demonstrated the intense staining of the thermogenic marker uncoupling protein 1 (UCP1) in subcutaneous WAT (scWAT, mouse SAT) [58]. Our ovarian granulosa cell tumor model which presented cachexia showed high staining of UCP1 in scWAT as well [59]. However, the protein expression of UCP1 remained unaltered in eWAT from the S2–013 orthotopic pancreatic cancer mice and VAT from stage IV PDAC patients [28]. Consistently, it was reported that Ucp1 expression remained unchanged in scWAT, and *Ucp1* mRNA expression rather decreased coincidently with *Pparg* mRNA expression in brown adipose tissue from Pan02-tumor-bearing mice [60]. Differential distribution of UCP1-positive cells in different adipose tissue has been demonstrated, and such work identifies scWAT as the most abundant source organ of UCP1-positive cells while little to no UCP1-positive cells are found in eWAT [61]. Therefore, because of the heterogeneity of cellular composition across different adipose tissue [62], the occurrence and contribution of adipocyte browning to adipose tissue loss would be limited to scWAT, but existing evidence regarding subcutaneous browning in a PC mouse model is inconsistent, requiring further investigation.

While PC cells lead to adipocyte metabolic dysfunction, it has been demonstrated that the gene expression of fibroblast-related markers is upregulated in adipocytes. Co-culture with PANC-1 significantly upregulated the expression of *S100a4*, *Acta2*, *Mmp-9*, and *Mmp-11* in 3T3-L1 adipocytes [50]. The protein expression of α-SMA and FSP1 was consistently found in 3T3-L1 adipocytes co-cultured with PANC-1 [51]. MIA PaCa2 co-culture also upregulated *S100a4*, *Aact2*, *Mmp11*, and *Col1a1* in 3T3-L1 adipocytes [50]. In this regard, we have reported that fibrotic remodeling prominently occurred in VAT from stage IV PDAC patients as demonstrated by intensive picrosirius red staining and immunofluorescence assay of collagen 1 and fibronectin [28]. As a potential signaling pathway mediating adipocyte phenotype shift to fibroblast-like cells, it was suggested that PC-derived WNT5a activates its downstream signaling molecules, c-Jun and AP1, with a demonstration that blocking WNT5a with either anti-WNT5a or WNT5a inhibitor (SFRP5) prevented adipocyte lipid depletion and its morphologic changes to fibroblast-like cells [54]. However, WNT5a is also expressed and secreted by mature adipocytes in adipose tissue and is rather shown to mediate adipocyte hypertrophy [63]. Therefore, further investigations are required regarding whether adipocyte dedifferentiation to the fibroblast-like cells would be translatable to the tissue fibrotic remodeling in vivo by identifying underlying mechanisms.

In humans, about 10% of existing adipocytes are replaced with new adipocytes annually in adipose tissue, and failure of adipocyte renewal would contribute to fibrotic remodeling of adipose tissue as a long-term consequence. New adipocytes are generated by a stepwise process, commonly referred to as adipogenesis [64]. Mesenchymal stem cells are abundantly found in adipose tissue, and its commitment to the adipose lineage produces preadipocytes [65]. Upon adipogenic hormonal stimulants, preadipocytes undergo growth arrest and subsequently express critical transcription factors for the differentiation process, such as PPARγ and CCAAT/enhancer binding protein alpha (C/EBPα) which induce adipocyte-specific genes [66]. In this regard, the pre-exposure to conditioned media from PANC-1 before the treatment with adipogenic stimulants significantly decreased 3T3-L1 adipocyte differentiation (Figure 2B), which is measured by oil red o staining that is widely used to detect the abundance of differentiated lipid-laden adipocytes [67]. Therefore, decreased PPARγ expression of adipose tissue in PC would implicate adipose tissue dysfunction that results from metabolic dysregulation in existing adipocytes but also suppressed adipocyte renewal.

## 5. Pancreatic Cancer-Associated Cachectic Mediators and Potential Mechanisms

There are several potential mediators that are PDAC-derived and mediate cachexia and adipose tissue loss. In PDAC patients, high circulating levels of Activin A, a homodimer of Inhibin βA subunits, are associated with poorer survival [28,68,69]. Our group demonstrated that high serum Activin A levels at diagnosis were correlated with a smaller size of adipocytes in VAT among stage IV PDAC patients [28]. In vivo studies suggest the existence of a direct relationship between Activin A and cachexia. Chen et al. [70] demonstrated that Activin A overexpression, by injecting AAV6 vectors carrying *Inhba* transgene into skeletal muscle tissues, induced weight loss in tumor-free mice. The weight loss mediated by Activin A overexpression was largely attributed to lean mass loss with a mild loss of fat mass in the model [70]. Walton et al. [71] also demonstrated that the implantation of Activin A-overexpressing Chinese Hamster Ovary (CHO) cell into quadriceps femoris muscle caused weight loss, but they found that fat mass loss was much greater than lean mass loss when compared to the mice implanted with control CHO cells. Furthermore, the subcutaneous injection of Activin A pro-peptide from the day after implantation rescued lean mass loss in the mice implanted with Activin A-overexpressing CHO, whereas fat mass loss was not prevented by the pro-peptide [71]. In KPC male mice, it was similarly demonstrated that the intraperitoneal injection of ACVR2B/Fc (antagonist for myostatin, Activin A, and growth differentiation factor 11 (GDF11)) prevented lean mass loss but showed no effects on fat mass loss [72]. On the other hand, ACVR2B/Fc failed to prevent lean mass loss and fat mass loss in KPC female mice [72]. Higher expression levels of endogenous Activin inhibitors such as *FSTL1* and *FSTL3* were suggested to play a role in driving sex difference in responses to ACVR2B/Fc [72]. Therefore, existing evidence supports the cachectic effects of Activin A on muscle wasting with a potential sex difference in Activin A-mediated cachexia in PDAC. However, more research is needed to address the role of Activin A in adipose tissue wasting.

Interleukin-6 (IL-6) is one of the inflammatory cytokines and forms a complex with a soluble or membrane-bound IL-6 receptor (IL-6R), which then binds to a transmembrane protein, glycoprotein 130, to induce intracellular signaling [73]. IL-6 is correlated with high serum free fatty acid levels in cachectic patients [74]. Additionally, IL-6 is shown to be elevated in PDAC patients [75]. The high staining of cancer cells for IL-6 on PDAC tumors is associated with the presence of cachexia in patients [76]. However, Ramsey et al. demonstrated that plasma IL-6 levels were not correlated with weight loss at diagnosis, but rather associated with the presence of metastasis in PDAC patients [77]. In mice, Rupert et al. [53] have shown that the orthotopic implantation of KPC cells significantly elevated plasma IL-6 levels and caused skeletal muscle and adipose tissue wasting, and such wasting in tissues was prevented in mice implanted with IL-6-deficient KPC cells. However, because tumor size was considerably suppressed in mice implanted with IL-6-deficient KPC cells, which would influence the degree of tissue wasting [53], they further provided in vitro evidence demonstrating that basal lipolysis in 3T3-L1 adipocytes was significantly promoted by soluble IL-6 receptor, not by exogenous IL-6 only. There are currently two on-going clinical trials testing the effects of IL-6R inhibitor on overall survival of PDAC patients during chemotherapy with or without immunotherapy (Identification numbers: NCT02866383 and NCT03193190). Therefore, clinical evidence is expected to provide a basis to address whether blocking IL-6 signaling would attenuate/reverse cachexia in PDAC patients.

Growth differentiation factor 15 (GDF15) has been suggested to mediate cancer-related cachexia because GDF15 has a high affinity to glial cell line-derived neurotrophic factor (GDNF) Family Receptor Alpha Like (GFRAL), which is highly expressed in hindbrain neurons, and the genetic ablation of GFRAL blunted the abilities of GDF15 to reduce food intake in mice [78,79]. The subcutaneous implantation of GDF15-overexpressing prostate cancer cell led to weight loss and reduced food intake in mice, whereas GDF15 antibody administration rescued the mice from weight loss especially by preventing lean mass wasting [80]. GDF15 levels were significantly elevated in tumors and sera from PDAC patients [81]. Still, limited evidence regarding the role of GDF15 in PC-related cachexia exists. It is assumed that GDF15 in PDAC might act as an anorexic mediator rather than directly targeting adipose and skeletal muscle tissues to mediate cachexia [82].

## 6. Conclusions

We have summarized the current literature in an attempt to understand the characteristics of PC-related cachexia. We highlight adipose tissue as the most affected organ in cachectic PC patients. Potential mechanisms include reduced intake, promoted fatty acid release, and fibrosis coincidently elevating free fatty acid and ketone levels in circulation (Figure 1). Additionally, we suggest decreased adipogenesis and promoted lipolysis in existing adipocytes underlying adipose atrophy during the development of PC-related cachexia (Figure 2C). However, much still remains to be learned about potential mediators and molecular mechanisms. Particular emphasis should thus be placed on identifying cachectic mediators and their mechanistic roles in mediating adipose atrophy in PC in the future.

## Data Availability

Not applicable.
